# Mycotoxins in Food: Cancer Risks and Strategies for Control

**DOI:** 10.3390/foods13213502

**Published:** 2024-10-31

**Authors:** Alice N. Mafe, Dietrich Büsselberg

**Affiliations:** 1 Department of Biological Sciences, Faculty of Sciences, Taraba State University, Main Campus, Jalingo 660101, Taraba State, Nigeria; mafealice1991@gmail.com; 2 Weill Cornell Medicine-Qatar, Education City, Qatar Foundation, Doha Metropolitan Area, P.O. Box 22104, Qatar

**Keywords:** mycotoxins, food, cancer risk, control strategies, analysis

## Abstract

Mycotoxins are toxic compounds produced by fungi such as *Aspergillus*, *Penicillium*, and *Fusarium*, contaminating various food crops and posing severe risks to food safety and human health. This review discusses mycotoxins‘ origins, significance, and impact, particularly in relation to cancer risk. Major mycotoxins like aflatoxins, ochratoxins, fumonisins, zearalenone, and patulin are examined, along with their sources and affected foods. The carcinogenic mechanisms of these toxins, including their biochemical and molecular interactions, are explored, as well as epidemiological evidence linking mycotoxin exposure to cancer in high-risk populations. The review also highlights critical methodologies for mycotoxin detection, including HPLC, GC-MS, MS, and ELISA, and the sample preparation techniques critical for accurate analysis. Strategies for controlling mycotoxin contamination, both pre- and post-harvest, are discussed, along with regulations from organizations like the FAO and WHO. Current challenges in detection sensitivity, cost, and control effectiveness are noted. Future research is needed to develop innovative analytical techniques, improve control strategies, and address the influence of climate change on mycotoxin production. Finally, global collaboration and emerging technologies are essential for advancing mycotoxin control and enhancing food safety.

## 1. Introduction

Mycotoxins are toxic secondary metabolites produced by various species of fungi, primarily molds, that pose significant risks to food safety and public health. These fungi, which include notable genera such as *Aspergillus*, *Penicillium*, and *Fusarium*, thrive on a wide range of food crops, especially under warm and humid conditions [1]. They can grow in the field, during harvest, and even during food storage, contaminating essential commodities like cereals, nuts, dried fruits, coffee, and spices. As a result, mycotoxins are a significant food safety concern, especially in regions where environmental conditions favor fungal growth and food preservation systems may be inadequate [2].

The production of mycotoxins by fungi is a natural defense mechanism, typically triggered under stressful conditions such as drought, insect damage, or improper food storage [3]. These toxic metabolites can contaminate food at any stage in the supply chain, from pre-harvest to processing and storage. Several mycotoxins are of particular concern due to their prevalence and toxicity. Aflatoxins, produced by *Aspergillus* species, are commonly found in peanuts, maize, and other grains and are well known for their carcinogenic properties [4]. Ochratoxins, produced by *Aspergillus* and *Penicillium* species, are often detected in cereals, coffee, and dried fruits [5]. Fumonisins, predominantly made by *Fusarium* species, are found in maize, while zearalenone and deoxynivalenol (DON), also produced by *Fusarium*, are common contaminants in wheat, barley, and corn [6].

The health risks posed by mycotoxins are significant. Some mycotoxins, such as aflatoxins, are highly carcinogenic and are directly linked to liver cancer, while others cause immunosuppression, kidney damage, and reproductive disorders [7]. Chronic exposure to low levels of mycotoxins can be detrimental to health. This challenge is enhanced in developing countries where food contamination is more prevalent and diets heavily depend on susceptible crops [8]. The health impacts are not limited to humans as livestock consuming contaminated feed may suffer, leading to economic losses in agriculture and food-producing sectors [9].

From an economic standpoint, mycotoxin contamination has far-reaching consequences. Contaminated crops may be rejected for sale, reducing yields and causing substantial financial losses for farmers and food producers [10]. Furthermore, the costs associated with mycotoxin detection, management, and control measures increase the economic burden, affecting local food security and international trade [11]. Countries with stricter food safety standards may reject imported contaminated food products, resulting in trade barriers that affect global food markets [12].

Due to mycotoxins’ health risks and economic impacts, regulatory bodies established stringent guidelines and limits for mycotoxin levels in food and animal feed [13]. International organizations like the Codex Alimentarius, in collaboration with the World Health Organization (WHO) and the Food and Agriculture Organization (FAO), set global standards aimed at minimizing mycotoxin contamination and protecting public health [14]. These regulatory efforts are critical in ensuring food safety, yet challenges remain in achieving comprehensive control across all stages of the food production process [15].

In summary, mycotoxins produced by molds that grow on various crops represent a significant concern in food safety due to their toxic effects on health, including the potential to cause cancer [16]. The economic implications of mycotoxin contamination further complicate food security and trade. Effective monitoring, control measures, and international regulatory standards are crucial in mitigating the impact of mycotoxins on public health and the global food supply chain [17].

Mycotoxins are crucial due to their significant impact on public health and food safety, particularly their association with cancer risk. Mycotoxins, such as aflatoxins, are highly potent carcinogens and are directly linked to liver cancer, among other health issues [18]. The International Agency for Research on Cancer (IARC) has classified aflatoxins as Group 1 carcinogens, indicating clear evidence of their cancer-causing potential in humans [19]. Such toxins, produced by fungi that contaminate staple food crops like maize, peanuts, and grains, can accumulate in the food chain, posing chronic health risks when consumed over time [20]. Therefore, regular food analysis and monitoring of mycotoxins are essential to prevent long-term exposure that could elevate cancer risks, particularly in vulnerable populations with limited dietary diversity [21].

The synergistic interactions between various mycotoxins, along with other environmental and dietary factors, significantly amplify their toxicity and complicate public health risks [22]. Multiple mycotoxins often co-occur in contaminated food or feed, such as aflatoxins and fumonisins in maize, where their combined presence enhances the carcinogenic potential beyond the effects of each toxin alone [23]. This synergism not only exacerbates liver cancer risk but also increases the likelihood of other adverse health outcomes, including immunosuppression and impaired growth [24]. Factors like dose and exposure levels further influence this synergism, where low doses of multiple mycotoxins can have enhanced effects than higher doses of a single one [25].

Metabolic interactions also play a role, with the metabolites of one mycotoxin potentially enhancing the toxic effects of another [26]. The immune system, often suppressed by certain mycotoxins, becomes more vulnerable to further toxic impact, while disruptions in gut microbiota and impaired detoxification processes can increase susceptibility to multiple mycotoxins. Nutritional deficiencies, particularly in essential nutrients like proteins, vitamins, and minerals, worsen these effects by reducing the body’s ability to detoxify mycotoxins, increasing vulnerability to chronic diseases, including cancer [27]. Additionally, mycotoxins can interfere with liver enzymes responsible for detoxification and induce oxidative stress, weakening the body’s defenses against toxins. Understanding these complex interactions is crucial for effective risk assessment, food safety interventions, and developing strategies to mitigate the health risks associated with multiple mycotoxin exposures [28].

In addition to cancer risks, mycotoxins present broader food safety concerns that necessitate thorough analysis. Contaminated foods can lead to a range of health issues beyond carcinogenicity, including immune suppression, gastrointestinal disorders, and reproductive problems [29]. These effects are particularly concerning in regions with poor food safety infrastructure, where contaminated foods may be widely consumed due to limited regulation or insufficient post-harvest management [30]. Ensuring food safety through analyzing mycotoxin levels helps mitigate these health risks and safeguard consumers and the global food supply chain [31]. Therefore, effective mycotoxin management is pivotal in food safety and cancer prevention and sustaining economic viability in the food industry [32].

### 1.1. Types of Mycotoxins

Different species of fungi produce common mycotoxins, which are a significant concern due to their toxic effects on human and animal health [33]. The most prevalent mycotoxins include aflatoxins, ochratoxins, fumonisins, zearalenone, and patulin, each with specific characteristics, sources, and health implications (Table 1) [34]. These mycotoxins contaminate a wide range of food products, making their presence a critical issue in food safety management.

Aflatoxins, ochratoxins, fumonisins, zearalenone, and patulin are the major mycotoxins of concern due to their prevalence in food products and significant health impacts. These toxins pose various risks, from carcinogenicity to reproductive and kidney disorders, emphasizing the need for rigorous monitoring and control in food production to ensure food safety.

### 1.2. Sources and Affected Foods of Mycotoxins

Mycotoxins contaminate various food products at multiple stages of production, from pre-harvest to post-harvest, due to fungal growth. Different fungi are responsible for producing these toxic compounds, with certain foods being more susceptible to contamination based on environmental conditions and storage practices [40]. Foods are commonly contaminated by major mycotoxins (Table 2) and the fungi responsible for their production.

**Figure 1:**
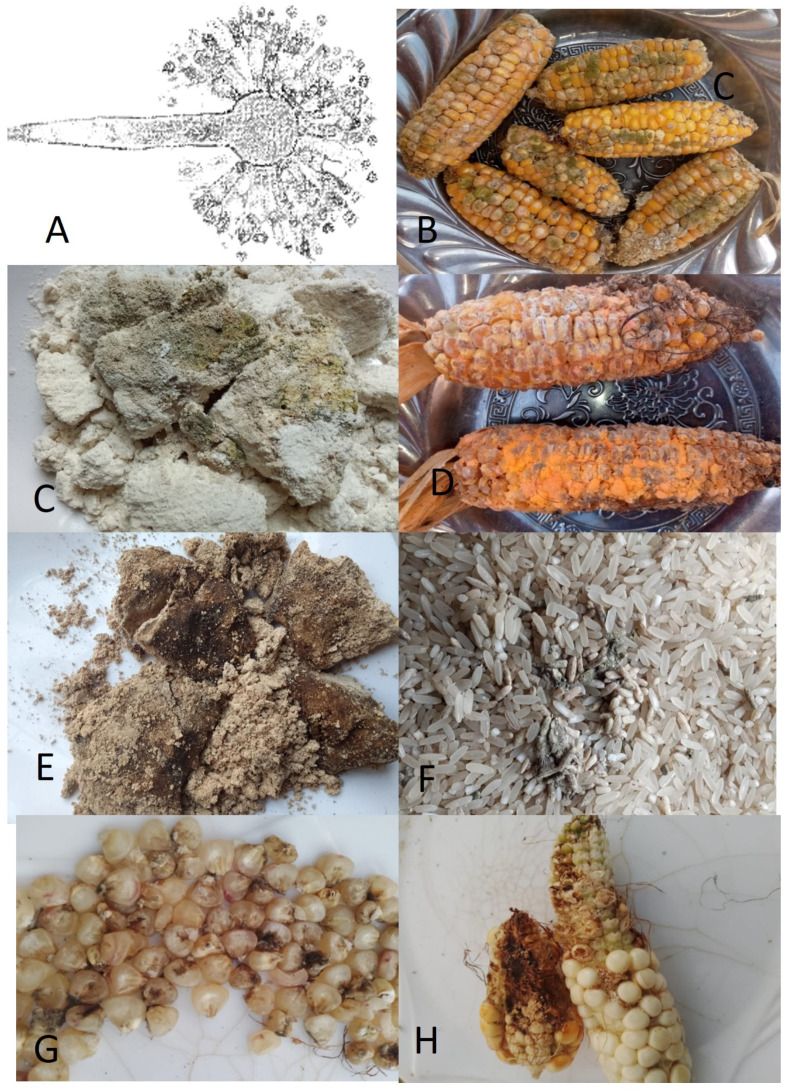
Molds generate mycotoxins in foods. (**A**) *Aspergilus fumigatus,* which releases aflatoxin, (**B**) molds on red corn, (**C**) molds on maize flour, (**D**) molds on red corn, (**E**) molds on soybean flour, (**F**) molds in rice grains, (**G**) mold on white maize grains, and (**H**) molds on white corn. All pictures by Alice N. Mafe.

Aflatoxins, ochratoxins, fumonisins, zearalenone, and patulin are the most concerning mycotoxins due to their prevalence in various food products and harmful health effects. The fungi producing these toxins thrive in specific environmental conditions, contaminating crops like maize, peanuts, cereals, dried fruits, and apples. Proper storage, handling, and monitoring of these foods are essential to reduce the risk of mycotoxin contamination and ensure food safety [45]. Table 3 provides an overview of the regulatory limits and health risk levels for various mycotoxins in food products, emphasizing the need for continuous monitoring and control to ensure food safety. Figure 1 represents the percentages for the estimated contribution of each mycotoxin to the total global mycotoxin burden based on toxicity and occurrence.

### 1.3. Global Toxic Levels of Mycotoxins in Foods

The global distribution of mycotoxins presents a significant concern for food safety due to their toxic effects on human and animal health. Based on global toxicity levels, aflatoxins are the most prevalent, accounting for 35% of the global toxic load. These highly toxic compounds are commonly found in crops like maize and peanuts, particularly in warm and humid climates, and are known for their carcinogenic properties [52].

Following aflatoxins, ochratoxin A and deoxynivalenol (DON) each contributes 20% to the total mycotoxin burden. Ochratoxin A is often found in cereals, coffee, and dried fruits and is associated with nephrotoxicity, while DON, commonly referred to as “vomitoxin”, occurs in grains and can cause acute gastrointestinal distress [53].

Fumonisins, which make up 15% of the global mycotoxin contamination, are prevalent in maize and are linked to esophageal cancer and neural tube defects. Lastly, zearalenone, responsible for 10% of the global burden, is an estrogenic mycotoxin found in grains that disrupt hormonal balance, particularly in livestock [34].

## 2. Cancer Risk Associated with Mycotoxins

Mycotoxins are widely recognized for their carcinogenic potential, with certain types posing significant cancer risks to humans. The primary mycotoxin associated with cancer risk is aflatoxin, which has been classified as a Group 1 carcinogen by the International Agency for Research on Cancer (IARC). Other mycotoxins, such as ochratoxin A and fumonisin B1, are also considered potential carcinogens. These toxins contribute to cancer development through various biochemical and molecular mechanisms that lead to genetic damage, cell cycle disruption, and immune suppression [54].

### 2.1. Mechanisms of Carcinogenicity

**Figure 2:**
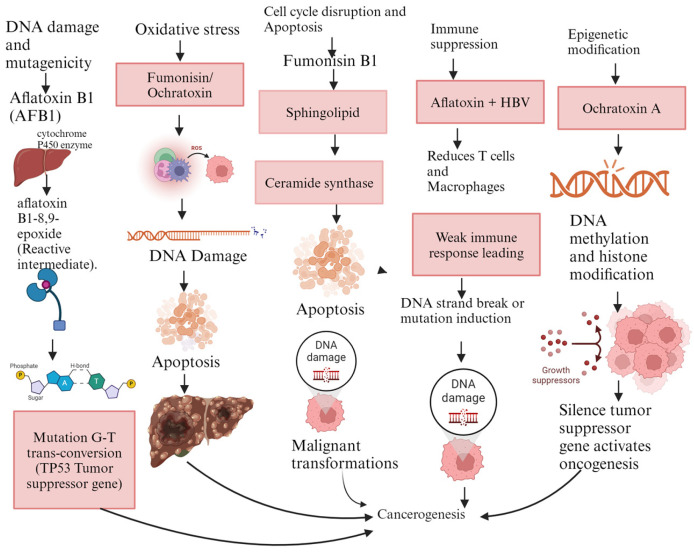
Mechanisms of carcinogenicity for aflatoxin B1, fumonisins, and ochratoxin A. Legend: AFB1: aflatoxin B1, HBV: hepatitis B virus, ROS: reactive oxygen species, TP53: tumor suppressor gene. The figure was generated using BioRender.

Mycotoxins, particularly aflatoxins, contribute to cancer development through multiple biochemical and molecular mechanisms. These include direct DNA damage and mutagenesis, oxidative stress, cell cycle regulation, disruption, apoptosis inhibition, immune suppression, and epigenetic modifications [55]. The cumulative effects of these mechanisms (Figure 2 and Table 4) can lead to the initiation, promotion, and progression of cancer, making mycotoxins a significant concern for public health, particularly in regions with high exposure to contaminated foods. Understanding these mechanisms is crucial for developing effective strategies to reduce the cancer risk associated with mycotoxin exposure [56].

### 2.2. Epidemiological Evidence Linking Mycotoxins to Cancer Risk

Epidemiological studies provide strong evidence linking exposure to mycotoxins, particularly aflatoxins, to an increased risk of cancer (Table 5). Most notably, aflatoxin exposure is extensively associated with liver cancer or hepatocellular carcinoma (HCC). Ochratoxin A and fumonisins have also been implicated in developing kidney and esophageal cancers, respectively. Below is an overview of critical studies and data illustrating the correlation between mycotoxin exposure and cancer risk (Table 5), focusing on high-risk populations and regions.

### 2.3. Cancer Risk Associated with Mycotoxins

Epidemiological evidence strongly supports the link between mycotoxin exposure and increased cancer risk, particularly liver cancer due to aflatoxins and esophageal and kidney cancers from fumonisins [72]. Populations in regions with high contamination and limited food safety measures are at risk. This underscores the urgent need for effective monitoring, regulatory measures, and food safety interventions to reduce exposure to mycotoxins and mitigate their associated cancer risks.

### 2.4. Non-Cancer Risks Associated with Aflatoxins

Aflatoxins, a group of mycotoxins produced by *Aspergillus* species, primarily *Aspergillus flavus* and *Aspergillus parasiticus*, are known for their carcinogenic properties. However, the non-cancer risks, particularly acute toxicities and their potential to cause stunting in infants, are significant public health concerns that merit further examination [41].

#### 2.4.1. Acute Toxicities of Aflatoxins

Aflatoxins can induce a range of acute toxic effects upon ingestion, particularly at high exposure levels. Acute aflatoxicosis is characterized by rapid-onset symptoms, which can vary depending on the dose and route of exposure. The main acute toxic effects include the following:

- Hepatotoxicity: The liver is the primary target organ for aflatoxins. Acute exposure can lead to liver damage, manifesting as jaundice, abdominal pain, and elevated liver enzymes. Severe cases can progress to liver failure, which may be fatal. The hepatotoxic effects are often attributed to the bioactivation of aflatoxins to reactive epoxide intermediates, leading to cellular damage and necrosis [73].
- Gastrointestinal Symptoms: Ingestion of contaminated food can cause gastrointestinal disturbances such as nausea, vomiting, abdominal cramps, and diarrhea. These symptoms result from direct irritation of the gastrointestinal tract and liver dysfunction.
- Immune System Suppression: Aflatoxins can impair immune function, making individuals more susceptible to infections. This is particularly concerning in infants, who already have immature immune systems. Immune suppression can lead to higher rates of morbidity and mortality from infectious diseases.
- Neurological Effects: In some cases, aflatoxin exposure has been linked to neurological symptoms, including headaches, confusion, and altered mental status. These effects may be due to hepatic encephalopathy resulting from liver dysfunction or direct neurotoxicity.

#### 2.4.2. Stunting in Infants

The relationship between aflatoxin exposure and stunting in infants is an emerging area of research, with significant implications for child health and development. Stunting refers to impaired growth and development in children, characterized by low height-for-age. It is a critical public health issue, as it can lead to long-term consequences for physical and cognitive development [74]. The mechanisms through which aflatoxin exposure may contribute to stunting include the following:

- Nutritional Deficiencies: Aflatoxins can interfere with nutrient absorption and metabolism. They can cause malabsorption syndromes by damaging the intestinal lining, leading to nutrient deficiencies, particularly of proteins, vitamins, and minerals essential for growth. Infants exposed to aflatoxins may not receive adequate nutrition, exacerbating the risk of stunting [75].
- Chronic Inflammation: Aflatoxin exposure can provoke an inflammatory response, resulting in chronic inflammation that impairs growth. Prolonged inflammation can alter metabolic processes and hinder the body’s ability to utilize nutrients effectively, which is critical for growth and development during infancy [76].
- Impaired Immune Function: As mentioned earlier, aflatoxins can suppress the immune system. Infants who experience repeated infections due to immune compromise may have increased metabolic demands and reduced nutrient absorption, contributing to stunting. Frequent illness can also lead to increased energy expenditure, diverting resources away from growth and development [77].
- Hormonal Disruption: Aflatoxins have been shown to affect the endocrine system, potentially disrupting growth hormone pathways. Any disruption in growth hormone signaling can have significant effects on growth and development, leading to stunted growth in infants [66].
- Maternal Exposure: The effects of aflatoxins are not limited to direct exposure in infants. Pregnant and lactating women exposed to aflatoxins can transfer these toxins to their infants through placental transfer and breast milk. This transference can adversely affect the growth and development of infants, compounding the risk of stunting [78].

#### 2.4.3. Public Health Implications

The non-cancer risks associated with aflatoxins, particularly acute toxicities and stunting in infants, highlight the urgent need for public health interventions. Effective strategies to mitigate aflatoxin exposure include the following:

- Food Safety Regulations: implementing strict regulations and monitoring systems to limit aflatoxin levels in food supplies, particularly in high-risk regions where staple crops are often contaminated [79].
- Education and Awareness: raising awareness among farmers, food processors, and consumers about the risks of aflatoxins, safe storage practices, and proper food handling techniques [80].
- Nutritional Interventions: providing nutritional support and supplementation for vulnerable populations, particularly in areas with high aflatoxin exposure, to mitigate the adverse effects of malnutrition and improve overall health outcomes.
- Research and Monitoring: continued research into the health effects of aflatoxins, particularly in children, and ongoing monitoring of aflatoxin levels in food sources will help to inform public health policies and interventions [81].

While aflatoxins are widely recognized for their carcinogenic properties, the acute toxicities and potential for stunting in infants represent significant non-cancer risks. Addressing these concerns is essential for improving child health and preventing long-term developmental consequences associated with aflatoxin exposure [82].

## 3. Methods of Analyzing Mycotoxins

Accurate detection and quantification of mycotoxins in food products are critical for ensuring food safety and preventing mycotoxin-related health risks, including cancer. Various analytical techniques are employed to identify and measure mycotoxins in agricultural products, processed foods, and animal feed. These methods differ in sensitivity, specificity, and complexity, allowing for qualitative and quantitative analysis across a range of mycotoxin types [83]. Table 6 describes the primary techniques used for mycotoxin analysis while Table 7 focuses on the recent publications on the immuno-detection of mycotoxins in food.

The analysis of mycotoxins in food relies on a combination of advanced techniques to ensure accurate detection and quantification. Chromatographic methods such as high-performance liquid chromatography (HPLC) and gas chromatography–mass spectrometry (GC-MS) are susceptible and precise, especially when combined with mass spectrometry [96]. Immunoassays, including enzyme-linked immunosorbent assay (ELISA) and lateral flow immunoassay (LFIA), offer rapid, cost-effective screening, making them suitable for routine testing [97]. Each method has its advantages depending on the type of mycotoxin, the complexity of the food matrix, and the desired level of accuracy, enabling comprehensive monitoring and control of mycotoxin contamination.

### Sample Preparation for Mycotoxin Analysis

Sample preparation is critical in accurately detecting and quantifying mycotoxins in food. The preparation process involves several stages aimed at isolating mycotoxins from complex food matrices while maintaining the integrity and concentration of the target mycotoxins [98]. Proper sample preparation reduces interference from food components, improves extraction efficiency, and enhances the sensitivity of subsequent analytical techniques [99]. The main steps in preparing food samples for mycotoxin analysis (Table 8) are sampling, homogenization, extraction, cleanup, and concentration.

Preparing food samples for mycotoxin analysis involves carefully executing steps to ensure accurate detection and quantification. Each stage, from sampling and homogenization to extraction, cleanup, and concentration, must be tailored to the specific type of food and mycotoxin being analyzed [102]. Proper sample preparation is essential for reducing interference, enhancing extraction efficiency, and improving analytical techniques’ reliability for mycotoxin detection.

## 4. Strategies for Mycotoxin Control

Controlling mycotoxin contamination is a pressing issue that requires a comprehensive approach. This approach should address pre- and post-harvest stages and incorporate effective regulatory and monitoring strategies. By implementing comprehensive control measures, we can significantly reduce the risk of mycotoxin contamination in food and feed, protecting public health and ensuring food safety [104].

### 4.1. Pre-Harvest Control Measures

Pre-harvest control measures focus on preventing fungal contamination and mycotoxin production before crops are harvested. These strategies involve various agricultural practices and interventions minimizing the conditions favoring fungal growth (Table 9) [62,75].

### 4.2. Post-Harvest Control Measures

Post-harvest control measures are crucial for minimizing mycotoxin contamination after harvesting. Proper storage conditions, such as maintaining cool and dry environments, are essential to inhibit fungal growth and mycotoxin production. Effective processing techniques, including cleaning, sorting, and milling, help reduce mycotoxin levels by removing contaminated parts and diluting mycotoxins. Chemical treatments, like ammonization and ozone treatment, can also detoxify mycotoxins in food products. Implementing these measures is vital to ensure food safety and protect public health.

### 4.3. Regulatory and Monitoring Approaches

Regulatory and monitoring approaches are essential for managing mycotoxin contamination and ensuring food safety. Regulatory bodies, such as the U.S. Food and Drug Administration (FDA) and the European Food Safety Authority (EFSA), establish guidelines and standards for maximum allowable levels of mycotoxins in food and feed [108]. These regulations protect public health by limiting exposure to mycotoxins through food and animal feed. Monitoring programs play a critical role in enforcing these standards. Regular testing of food and feed samples for mycotoxin contamination helps to identify and mitigate risks before they reach consumers. Techniques such as high-performance liquid chromatography (HPLC) and enzyme-linked immunosorbent assays (ELISAs) are commonly used to ensure compliance with regulatory limits. Additionally, government agencies and industry organizations often collaborate to conduct surveillance and train producers on best practices for managing mycotoxin risks [109].

#### Mycotoxin Regulation Framework

Global regulatory limits for various mycotoxins are set by different countries and international bodies, highlighting the variation in food safety standards. Aflatoxins, known for their carcinogenic effects, are regulated most strictly in the European Union (EU) at 2 ppb, while the United States (FDA) allows up to 20 ppb. Ochratoxin A, a nephrotoxic mycotoxin, has a 5 ppb limit in most regions, though it is unregulated in the U.S. [14]. For fumonisins, the limits range widely, from 1000 ppb in China to 4000 ppb in the U.S. Zearalenone, which affects hormone regulation, has limits varying from 60 ppb in China to 200 ppb in Japan, with no regulation in some countries. Lastly, deoxynivalenol (DON), known for its gastrointestinal effects, is regulated between 1000 ppb in the U.S. and 2000 ppb in Australia/New Zealand [110]. These data provide a basis for comparing the regulatory frameworks, emphasizing the need for harmonized global standards to ensure food safety.

## 5. Current Challenges and Limitations

The effective management of mycotoxin contamination in food and feed presents several challenges and limitations, particularly concerning detection methods and control strategies. Resolving these issues is crucial for improving food safety and mitigating health risks associated with mycotoxins.

### 5.1. Detection Challenges

Detection of mycotoxins in food involves sophisticated analytical techniques that face several challenges, including sensitivity, specificity, and cost (Table 10), while Table 11 illustrates the various regulatory and monitoring approaches. One significant challenge is achieving the required sensitivity to detect low levels of mycotoxins, especially when they are present in complex food matrices. Techniques such as high-performance liquid chromatography (HPLC) and gas chromatography–mass spectrometry (GC-MS) offer high sensitivity but can be expensive and require extensive sample preparation [111]. In addition, the specificity of these methods must be high to accurately distinguish between mycotoxins and similar compounds that may interfere with results.

Another challenge is the cost of advanced detection methods, which may be prohibitive for routine testing in low-resource settings. While immunoassays like enzyme-linked immunosorbent assays (ELISAs) are more cost-effective, they may lack the sensitivity and specificity required for detecting low levels of mycotoxins in complex samples [118]. Furthermore, developing and validating new detection methods can be time-consuming and resource-intensive.

### 5.2. Control Measures Limitations

While various control strategies are employed to manage mycotoxin contamination, each has its limitations in terms of effectiveness and feasibility. 

Managing mycotoxin contamination involves addressing several challenges related to detection and control measures. Detection methods must be sensitive and specific, but high costs and technical limitations can restrict their use. Control measures, including pre-harvest practices, post-harvest treatments, and chemical decontamination, face limitations in effectiveness and feasibility. Regulatory and monitoring systems are crucial for ensuring compliance but may encounter consistency and resource availability challenges. To improve food safety and mitigate mycotoxin risks, ongoing research, innovation, and investment in more effective and accessible methods are needed [119].

## 6. Future Directions

The management and control of mycotoxins continue to evolve as new challenges and opportunities arise. Further research and the adoption of emerging technologies are essential to address these issues effectively. Identifying research gaps and staying abreast of emerging trends, such as advanced biosensors, machine learning models, and genome editing tools, can help enhance our ability to detect, control, and mitigate the risks associated with mycotoxins. This proactive approach ensures that food safety strategies remain dynamic and effective in the face of evolving threats [120].

### 6.1. Research Gaps

In recent years, significant progress has been made in understanding mycotoxins and their impact on food safety. However, several critical research gaps remain, hindering the development of more effective detection, control, and management strategies. Table 12 highlights these gaps, the needs they address, and the opportunities for future research to improve food safety and mitigate the risks posed by mycotoxins [121]

### 6.2. Emerging Trends

As mycotoxin management evolves, several emerging trends are reshaping how mycotoxins are detected, controlled, and regulated. These advancements are driven by technological innovations, research into sustainable solutions, and enhanced global cooperation. Table 13 outlines the key emerging trends, highlighting their potential impact on food safety, agricultural practices, and regulatory frameworks [7]. In addition, Table 14 presents the research gaps, while Table 15 discusses key emerging trends.

Addressing the challenges associated with mycotoxins requires ongoing research and adopting new technologies. Identifying research gaps, such as the need for advanced analytical methods and innovative control strategies, is crucial for improving mycotoxin management [113]. Emerging trends, including advancements in analytical technology, the integration of AI and ML, and the development of biocontrol agents, offer promising opportunities to enhance detection and control efforts [136]. By staying informed about these developments and investing in research and innovation, we can better manage the risks associated with mycotoxins [137] and protect public health.

## 7. Conclusions

This review examined the complex issue of mycotoxins in food, emphasizing their origin, significance, and impact on public health. Mycotoxins, toxic secondary metabolites produced by fungi such as Aspergillus, Penicillium, and Fusarium, represent a severe threat to food safety and public health. Key mycotoxins of concern include aflatoxins, ochratoxins, fumonisins, zearalenone, and patulin, each with specific sources and affected foods. The cancer risk associated with mycotoxins is notably significant, with aflatoxins being particularly carcinogenic and linked to liver cancer. Epidemiological evidence highlights a heightened risk in areas with substantial mycotoxin exposure, often exacerbated by inadequate control measures and regulatory oversight. Detection of mycotoxins remains challenging due to analytical methods’ sensitivity, specificity, and cost issues. While techniques such as chromatography and immunoassays are effective, they have limitations that affect their reliability and accessibility. Control measures, including pre-harvest and post-harvest strategies, along with regulatory frameworks, are crucial but have limitations regarding feasibility and effectiveness. Emerging trends, such as advancements in analytical technology, the integration of AI and machine learning, and the development of biocontrol agents, offer promising opportunities for enhancing mycotoxin management. However, research gaps persist, particularly in developing new methods, understanding mycotoxin interactions, and adapting to climate change. To improve the management of mycotoxin contamination and ensure food safety, it is recommended to invest in advanced analytical technologies, develop and implement innovative control strategies, address research gaps and emerging risks, strengthen regulatory and monitoring frameworks, and promote global collaboration and data sharing. Addressing these challenges requires a coordinated effort that includes advancing technology, enhancing control measures, and fostering international cooperation to manage mycotoxin risks and protect public health effectively.

## Figures and Tables

**Table 1:** Types of mycotoxins in foods.

Mycotoxins	Description
Aflatoxins	Aflatoxins are among the most studied and dangerous mycotoxins, primarily produced by *Aspergillus flavus* and *Aspergillus parasiticus* species. They commonly contaminate crops such as maize, peanuts, and tree nuts, especially in warm, humid climates. Aflatoxins are highly toxic and carcinogenic, with aflatoxin B1 being the most potent. Chronic exposure to aflatoxins has been linked to liver cancer, particularly in regions with high consumption of contaminated grains. Aflatoxins can also cause acute poisoning (aflatoxicosis), liver damage, immune suppression, and stunted growth in children. Due to their severe health effects, aflatoxins are highly regulated globally in food and feed products [35].
Ochratoxins	Ochratoxins, particularly ochratoxin A, are produced by species of *Aspergillus* and *Penicillium* and are commonly found in cereals, dried fruits, coffee, and wine. Ochratoxin A is nephrotoxic, meaning it can cause damage to the kidneys, and is also considered a potential carcinogen. Long-term exposure to ochratoxins has been associated with kidney disorders, such as Balkan Endemic Nephropathy, and may also have immunosuppressive effects. Ochratoxin contamination often occurs during improper food storage, especially in humid conditions, making post-harvest management crucial in preventing its occurrence [36].
Fumonisins	Fumonisins are primarily produced by *Fusarium* species, with *Fusarium verticillioides* being a common contaminant of maize. These mycotoxins are particularly prevalent in regions where maize is a dietary staple. Fumonisin B1 is the most toxic form, and it is associated with a range of health effects, including esophageal cancer, neural tube defects, and liver and kidney toxicity. In animals, fumonisins have been linked to diseases such as equine leukoencephalomalacia (ELEM) in horses and pulmonary edema in pigs. Controlling fumonisin contamination is vital to reduce both human and animal health risks [37].
Zearalenone	Zearalenone is another mycotoxin produced by *Fusarium* species, often found in maize, wheat, and barley. Zearalenone mimics estrogen in animals and humans, making it a significant concern for reproductive health. Exposure to zearalenone can cause reproductive disorders, including infertility, in livestock and may also disrupt hormonal balance in humans. It is particularly problematic in livestock feed, leading to economic losses in animal husbandry. While not classified as a potent carcinogen, zearalenone’s endocrine-disrupting effects highlight the importance of controlling its levels in food and feed [38].
Patulin	Patulin is a mycotoxin primarily produced by *Penicillium* and *Aspergillus* species and is most commonly associated with moldy fruits, particularly apples. Contamination by patulin can occur during the production of fruit juices, mainly when damaged or decayed fruits are processed. Although patulin is not a potent carcinogen, it can cause gastrointestinal distress and is mutagenic in certain studies. Regulations limit patulin levels in fruit products, particularly apple-based foods, to protect consumers from its toxic effects [39].

**Table 2:** Major mycotoxins in several food items.

Mycotoxin	Responsible Fungi	Affected Foods
Aflatoxins	Aflatoxins are mainly produced by *Aspergillus flavus* and *Aspergillus parasiticus*. These fungi thrive in warm and humid environments, making aflatoxin contamination more common in tropical and subtropical regions.	Aflatoxins commonly contaminate maize (corn), peanuts, tree nuts (almonds, pistachios, and walnuts), cottonseed, and some spices. Improper storage conditions, particularly in humid environments, increase the likelihood of aflatoxin contamination in these crops. Additionally, dairy products can be affected by aflatoxin M1 when livestock consume contaminated feed [41].
Ochratoxins	Ochratoxins, particularly ochratoxin A, are produced by species of *Aspergillus* (notably *Aspergillus ochraceus*) and *Penicillium* (*Penicillium verrucosum*).	Ochratoxins are commonly found in cereals such as wheat, barley, and oats, as well as in coffee, dried fruits (like raisins and figs), wine, beer, and grape juice. Contamination often occurs in stored grains, particularly under poor storage conditions with high moisture levels. Additionally, ochratoxins have been found in spices and cured meats [42].
Fumonisins	Fumonisins are produced primarily by *Fusarium verticillioides* (formerly *Fusarium moniliforme*) and *Fusarium proliferatum*, widespread in maize-growing regions.	Fumonisins are most commonly associated with maize (corn) and its products, including cornmeal, popcorn, cornflakes, and animal feed made from corn. In regions where maize is a dietary staple, fumonisin contamination is particularly concerning. The toxin may also be found in other cereal grains like sorghum [43].
Zearalenone	Zearalenone is produced by *Fusarium graminearum* and *Fusarium culmorum and is* commonly found in temperate climates.	This mycotoxin frequently contaminates maize, wheat, barley, oats, and rye. It is also commonly found in animal feed, disrupting reproductive functions in livestock, particularly pigs. Zearalenone contamination often occurs during harvest or storage when grains are exposed to moist conditions [44].
Patulin	Patulin is mainly produced by *Penicillium expansum*, and some species of *Aspergillus* and *Byssochlamys*. It grows primarily on decaying or damaged fruits.	Patulin is most found in apples and apple-derived products, such as apple juice and cider, mainly when damaged or moldy apples are processed. Other fruits that may be contaminated with patulin include pears, peaches, grapes, and apricots. Contamination can also occur in fruit juices and jams if compromised fruit is used during processing [39].

**Table 3:** Toxic levels of various mycotoxins in food.

Mycotoxin	Toxic Level in Food	Food Sources
Aflatoxins	Maximum allowable limit: 4 µg/kg (EU regulation).	Nuts, grains, corn, and spices.
	Toxic level associated with health risks: 0.5 µg/kg [46].	Peanuts, tree nuts, and maize.
Zearalenone	Maximum allowable limit: 100 µg/kg (EU regulation).	Cereals, grains, maize, and animal feed.
	Toxic level associated with health risks: 50 µg/kg [47].	Maize products, wheat, barley.
Patulin	Maximum allowable limit: 50 µg/kg (EU regulation).	Apples, apple juice, and apple-based products.
	Toxic level associated with health risks: 25 µg/kg.	Processed fruit products.
Fumonisins	Maximum allowable limit: 4000 µg/kg (EU regulation) [48].	Corn and corn-based products.
	Toxic level associated with health risks: 2000 µg/kg.	Maize and maize flour.
Ochratoxin A	Maximum allowable limit: 3 µg/kg (EU regulation).	Coffee, cereals, and dried fruit.
	Toxic level associated with health risks: 1 µg/kg [49].	Wine, grains, and legumes.
Deoxynivalenol (DON)	Maximum allowable limit: 1750 µg/kg (EU regulation).	Wheat, barley, and oats.
	Toxic level associated with health risks: 1000 µg/kg [50].	Cereal products and animal feed.
T-2 Toxin	Maximum allowable limit: 1000 µg/kg (EU regulation).	Cereal grains and animal feed.
	Toxic level associated with health risks: 500 µg/kg [51].	Wheat, barley, and oats.

**Table 4:** Mechanisms of carcinogenicity caused by mycotoxins.

Mechanism/Toxin	Description
DNA Damage and Mutagenicity	Aflatoxins, particularly aflatoxin B1 (AFB1), are potent carcinogens that exert their effects by directly damaging DNA. Once ingested, AFB1 is metabolized in the liver by cytochrome P450 enzymes into a reactive intermediate, aflatoxin B1-8,9-epoxide. This metabolite can bind covalently to DNA, forming DNA adducts, particularly at the guanine base, leading to mutations. One of the most common mutations caused by AFB1 is the G-to-T transversion in the TP53 tumor suppressor gene, which is crucial in regulating cell growth and apoptosis. Mutations in TP53 result in uncontrolled cell proliferation and are strongly associated with hepatocellular carcinoma (liver cancer). Aflatoxin-induced DNA damage is thus a key mechanism driving the initiation of cancer [57].
Oxidative Stress	Mycotoxins can also induce oxidative stress, a condition where there is an imbalance between the production of reactive oxygen species (ROS) and the body’s ability to detoxify them. Aflatoxins and other mycotoxins, such as fumonisins and ochratoxins, can generate ROS during their metabolism, leading to oxidative damage to cellular components like DNA, proteins, and lipids. This oxidative damage can cause mutations, promote inflammation, and contribute to the initiation and progression of cancer. In addition, chronic oxidative stress can disrupt cellular signaling pathways that control cell growth and apoptosis, further promoting carcinogenesis [58].
Cell Cycle Disruption and Apoptosis Inhibition	Mycotoxins can interfere with normal cell cycle regulation, contributing to the development of cancer. For instance, fumonisin B1, commonly found in maize, disrupts sphingolipid metabolism by inhibiting ceramide synthase. Sphingolipids are essential in regulating cell growth, differentiation, and apoptosis. The disruption of sphingolipid pathways can impair apoptosis (programmed cell death), allowing damaged cells to survive and proliferate uncontrollably, a hallmark of cancer development. Additionally, by blocking apoptosis, mycotoxins facilitate the survival of cells with DNA damage, increasing the likelihood of malignant transformation [59].
Immune Suppression	Chronic exposure to certain mycotoxins can lead to immune suppression, which further increases cancer risk. Aflatoxins, for example, are known to impair the immune system by reducing the production and function of immune cells like T-cells and macrophages. This weakened immune response hampers the body’s ability to recognize and eliminate cancerous or pre-cancerous cells. Furthermore, immune suppression can promote the persistence of viral infections, such as hepatitis B virus (HBV), which is a significant cofactor in aflatoxin-induced liver cancer. Individuals who are exposed to both aflatoxins and HBV are at a much higher risk of developing liver cancer due to the combined effects of viral infection and toxin-induced DNA damage [60].
Epigenetic Modifications	In addition to directly damaging DNA, mycotoxins can cause epigenetic changes that alter gene expression without affecting the underlying DNA sequence. For instance, mycotoxins like ochratoxin A have been shown to induce changes in DNA methylation and histone modifications, which can silence tumor suppressor genes or activate oncogenes. These epigenetic alterations can promote carcinogenesis by disrupting normal cellular functions and facilitating uncontrolled cell growth [61].
Zearalenone (ZEA)	Zearalenone is a nonsteroidal estrogenic mycotoxin primarily produced by Fusarium species, commonly found in cereals and grains. The carcinogenicity of ZEA is primarily linked to its estrogenic properties, as it mimics the action of natural estrogens by binding to estrogen receptors (ERs) in target tissues. This interaction leads to hormonal disruption, which promotes the proliferation of estrogen-sensitive cells, particularly in reproductive tissues. Over time, the hyperproliferation of these cells increases the risk of hormone-dependent cancers, such as breast, ovarian, and endometrial cancers. ZEA’s ability to activate ER signaling can also induce DNA damage and oxidative stress, further contributing to its carcinogenic potential. Oxidative stress generates reactive oxygen species (ROS), which can cause mutations, impair DNA repair mechanisms, and lead to genomic instability. Additionally, ZEA may disrupt normal cell cycle regulation, promoting abnormal cell division and enhancing the risk of cancer development [62].
Patulin	Patulin, produced by Penicillium and Aspergillus species, is a mycotoxin primarily found in apples and apple products. Its carcinogenicity is associated with its ability to induce oxidative stress and DNA damage. Patulin promotes the generation of ROS, which can damage cellular components, including lipids, proteins, and nucleic acids. This oxidative damage leads to mutations and chromosomal aberrations, increasing the risk of malignant transformations. Furthermore, patulin interferes with key cellular pathways involved in apoptosis (programmed cell death) and cell cycle regulation. By inhibiting apoptosis, patulin allows damaged cells to survive and proliferate, which may contribute to cancer initiation and progression. Patulin also impairs the function of tumor suppressor proteins, such as p53, which generally help to maintain genomic integrity by halting the cell cycle in response to DNA damage. When p53 function is disrupted, cells with damaged DNA can continue to divide uncontrollably, further contributing to the carcinogenic process [63].

**Table 5:** Key studies and data illustrating the correlation between mycotoxin exposure and cancer risk for zearalenone and patulin.

Mycotoxin	Findings	Cancer Type
Zearalenone	Reported increased estrogenic activity of zearalenone in human breast cancer cells, leading to cell proliferation [64].	Breast Cancer
	Found that dietary exposure to zearalenone in rats led to a significant increase in uterine weight and hyperplasia [65].	Uterine Cancer
	Identified a link between zearalenone exposure and increased risk of reproductive cancers through hormonal disruption [66].	Reproductive Cancers
	Showed that zearalenone exposure caused oxidative stress and DNA damage in liver cells, potentially increasing liver cancer risk [67].	Liver Cancer
Patulin	Documented the DNA-damaging effects of patulin in human liver cells, leading to mutagenic changes [68].	Liver Cancer
	Investigated the carcinogenic potential of patulin in mouse models, noting an increase in tumor incidence [69].	Multiple Cancer Types
	Found that patulin exposure induced oxidative stress and apoptosis in colon cancer cells, highlighting its potential role in colorectal cancer [70].	Colorectal Cancer
	Reported that dietary exposure to patulin in rats resulted in liver toxicity and increased cancer risk [71].	Liver Cancer

**Table 6:** Primary techniques used for mycotoxin analysis.

Method	Description		
Chromatography	Chromatography is one of the most widely used techniques for analyzing mycotoxins due to its high sensitivity, accuracy, and ability to separate and identify multiple mycotoxins simultaneously. Two common forms of chromatography used in mycotoxin analysis are high-performance liquid chromatography (HPLC) and gas chromatography–mass spectrometry (GC-MS) [84].		
	Principle	Application	Advantage
High-Performance Liquid Chromatography (HPLC)	HPLC involves the separation of mycotoxins based on their interaction with a stationary phase (usually a column) and a mobile phase (usually a solvent). The different affinities of mycotoxins for the stationary phase allow them to be separated, detected, and quantified [85].	HPLC is commonly used to analyze aflatoxins, fumonisms in various foods, including cereals, nuts, and dairy products. It is highly effective when coupled with fluorescence or UV detection methods, which enhance sensitivity for specific mycotoxins.	HPLC offers high resolution, accuracy, and the ability to detect low levels of mycotoxins. It is widely accepted in regulatory testing and can be used for routine food safety monitoring.
Gas Chromatography–Mass Spectrometry (GC-MS)	GC-MS vaporizes mycotoxin samples, separates them via gas chromatography, and identifies them by mass spectrometry. Mycotoxins are derivatized to ensure volatility [86].	GC-MS is beneficial for the detection of volatile mycotoxins like patulin. It is susceptible and specific, making it suitable for detecting trace levels of mycotoxins in complex food matrices.	GC-MS provides high specificity and sensitivity, making it the gold standard for detecting mycotoxins like patulin in fruit juices.
Spectrometry	Mass spectrometry (MS) is often combined with chromatography to improve the sensitivity and specificity of mycotoxin detection. MS measures the mass-to-charge ratio of ionized mycotoxin molecules, providing precise molecular identification and quantification.		
Mass Spectrometry (MS)	MS works by ionizing chemical compounds and measuring the mass-to-charge ratio of the resulting ions. Coupled with HPLC or GC, it allows for separating and identifying mycotoxins based on their mass [87].	HPLC-MS and GC-MS are widely used to analyze various mycotoxins, including aflatoxins, ochratoxins, and fumonisins. These techniques are valuable in multi-mycotoxin analysis, where several toxins may exist in a single sample.	MS provides high accuracy and detects multiple mycotoxins at low concentrations, which is crucial for regulatory testing and detailed mycotoxin profiling in food products.
Immunoassays	Immunoassays are rapid, sensitive, and cost-effective techniques for detecting mycotoxins in food. They rely on antibodies’ specific binding to mycotoxins and are suitable for quickly screening large numbers of samples [88].		
Enzyme-Linked Immunosorbent Assay (ELISA)	ELISA is based on antibodies binding to mycotoxins, followed by an enzyme–substrate reaction that produces a detectable signal, usually colorimetric or fluorescent. The intensity of the signal corresponds to the concentration of mycotoxins in the sample [89].	ELISA is commonly used to detect aflatoxins, ochratoxns, zearalenone, and fumonisms in food products such as grains, nuts, and milk. It is often employed for routine screening in food industries and regulatory bodies.	ELISA is a quick, affordable method for mycotoxin detection but may lack the specificity of chromatographic techniques due to cross-reactivity.
Lateral Flow Immunoassay (LFIA)	LFIA is similar to ELISA but uses a test strip format. Mycotoxin–antibody interactions produce a visible line or signal on the test strip, indicating the presence of mycotoxins [90].	LFIA is used for rapid, on-site testing of mycotoxins in agricultural products. It is commonly applied to detect aflatoxins, fumonisins, and zearalenone in grains, nuts, and animal feed.	LFIA is a portable, quick method for mycotoxin detection, ideal for field testing, though less precise than lab methods.
Hyperspectral Analysis	Utilizes the spectral signature of materials across a wide range of wavelengths to identify and quantify mycotoxin contamination [91].	Sorting and detecting mycotoxin presence in grains, nuts, and other food products.	Non-destructive, rapid analysis, can be applied in real-time sorting, and high-throughput screening.
Immuno-detection	Employs specific antibodies that bind to mycotoxins, allowing their detection through various methods (e.g., ELISA, lateral flow assays) [90].	Food safety testing, monitoring mycotoxin levels in processed and raw food products.	High specificity and sensitivity, can detect low concentrations of mycotoxins, and suitable for various matrices.

**Table 7:** Recent publications on immuno-detection of mycotoxins.

Technique	Findings
ELISA	Developed a novel ELISA method for detecting aflatoxins in peanuts with high sensitivity [92].
Lateral Flow Immunoassay	Created a lateral flow immunoassay for rapid detection of zearalenone in cereal products [93].
Immunoaffinity Columns	Utilized immunoaffinity columns for the extraction and detection of patulin in fruit juices, achieving high recovery rates [94].
Magnetic Nanoparticles	Developed magnetic nanoparticles coupled with immunoassays for the detection of multiple mycotoxins in grains [95].

**Table 8:** Main steps in preparing food samples for mycotoxin analysis.

Sample Preparation	Importance	Procedure	Challenges
Sampling	Accurate mycotoxin analysis relies on proper sampling, as uneven distribution in food can cause incorrect results.	The process begins with collecting food samples from different parts of a batch to account for variability in mycotoxin contamination. To form a composite sample, random sub-samples are collected and combined for solid foods like grains, nuts, and cereals. Mixing ensures even distribution before sampling for liquids, milk, or fruit juices [100].	Heterogeneous mycotoxin contamination complicates sampling, requiring larger samples for bulk goods like grains to minimize errors.
Homogenization	After sampling, food must be homogenized to ensure even distribution of mycotoxins, particularly in solid or semi-solid foods [101].	Homogenization involves grinding or blending the sample into a fine, uniform consistency. Equipment such as mills or blenders reduces the particle size of solid foods like grains or nuts. Mixing ensures consistency in liquids.	Prevent contamination during homogenization, as excessive grinding heat can degrade sensitive mycotoxins.
Extraction	The extraction aims to separate mycotoxins from the food matrix into a solvent, isolating them from interfering compounds like proteins, fats, and carbohydrates for easier analysis.	The solvent choice depends on the food type and mycotoxin analyzed. Common organic solvents like methanol and acetonitrile effectively dissolve mycotoxins. The homogenized food is mixed with the solvent and agitated for solid samples, while liquid samples require filtration or centrifugation to remove debris before analysis [102].	The selection of an appropriate solvent system is crucial. It must efficiently extract mycotoxins while minimizing the coextraction of other food components that may interfere with the analysis.
Cleanup	Following extraction, a cleanup step is often necessary to remove unwanted compounds from the extract, such as fats, sugars, and proteins, which can interfere with the sensitivity and accuracy of detection methods.	Cleanup methods vary by analytical technique and food matrix:Solid-Phase Extraction (SPE): extracts pass through an adsorbent column that binds unwanted substances while allowing mycotoxins to pass.Immunoaffinity Columns (IAC): use antibodies to selectively bind and isolate mycotoxins, which are then eluted with a solvent.Liquid–Liquid Partitioning: separates mycotoxins based on solubility in two immiscible phases, typically an organic solvent and water [103].	Cleanup must be optimized for different food types, as excessive removal of matrix components can result in the loss of mycotoxins, reducing the sensitivity of the analysis.
Concentration	After extraction and cleanup, mycotoxin levels may be too low for accurate detection. Concentration improves these levels, making quantification easier.	Concentration is typically achieved by evaporating the solvent used during extraction, leaving behind a more concentrated sample of mycotoxins. This is usually performed under reduced pressure or using rotary evaporation to avoid degradation of the mycotoxins [98].	Avoid over-concentration, as it can cause matrix effects or unwanted compound precipitation that interferes with analysis.

**Table 9:** Strategies involve various agricultural practices and interventions minimizing the conditions favoring fungal growth.

	Principle	Implementation	Challenges
Crop Rotation	Crop rotation alternates crops in a field across seasons, reducing fungal populations that target specific crops and lowering mycotoxin contamination risk [105].	By rotating crops, such as alternating cereals with legumes or other non-host plants, the life cycle of fungal pathogens is disrupted, reducing their ability to infect subsequent crops.	Effective crop rotation requires careful planning to prevent new crops from hosting pathogens and adapting practices to local conditions.
Use of Resistant Varieties	Plant breeding programs focus on developing crop varieties resistant to specific fungal pathogens. Resistant varieties can reduce fungal infection and subsequent mycotoxin production [106].	Farmers can select and plant varieties of crops that have been genetically modified or selectively bred for resistance to mycotoxin-producing fungi, such as maize varieties resistant to *Fusarium* species.	Regional factors may limit the availability of resistant varieties, and continuous breeding efforts are needed to address evolving fungal strains. Moreover, resistance does not always guarantee complete protection, so it should be used in conjunction with other measures.
Proper Irrigation and Field Management	Fungal pathogens thrive in warm, humid conditions, making proper irrigation and field management critical in preventing fungal contamination [107].	Techniques include optimizing irrigation, ensuring good drainage, and avoiding over-fertilization to reduce fungal growth. Managing plant residue and minimizing mechanical damage also help prevent fungal infection.	Effective field management requires monitoring weather conditions, soil moisture levels, and crop health, which may be resource-intensive. Farmers need access to proper tools and training to implement these practices effectively.

**Table 10:** Post-harvest control measures.

Measure	Principle	Implementation	Challenges
Storage Conditions	Proper storage conditions are vital for preventing fungal growth and mycotoxin production post-harvest [112].	Critical practices include maintaining dry, cool, and ventilated storage to prevent fungal growth. Grains should be stored below 14% moisture in ventilated containers, with regular inspections of storage facilities.	Maintaining optimal storage conditions requires ongoing monitoring and control, which can be challenging in regions with limited infrastructure or resources.
Processing Techniques	Processing methods can help reduce mycotoxin levels in food products and remove contaminated portions [113].	Techniques like cleaning and sorting can remove contaminated food parts. Heat treatments may degrade some mycotoxins, while good manufacturing practices (GMPs) and hazard analysis and critical control point (HACCP) systems help manage contamination risks.	The effectiveness of processing techniques depends on the type of mycotoxin and the food matrix. Not all mycotoxins are easily removed or degraded by processing methods.
Chemical Treatments	Chemical treatments can help neutralize or remove mycotoxins from food and feed [114].	Adsorbents like activated carbon or clay can be added to animal feed to bind mycotoxins and reduce their bioavailability. Chemical decontamination agents, such as ozone or ammonia, can be used to treat contaminated grains.	Chemical treatments should be used cautiously to prevent new contaminants and preserve nutritional quality, as their effectiveness varies by mycotoxin type and treatment method.

**Table 11:** Regulatory and monitoring approaches.

Approach	Principle	Implementation	Challenges
Regulatory Guidelines and Standards	Regulatory guidelines establish permissible mycotoxin levels in food and feed to safeguard public health.	International bodies like Codex Alimentarius set global mycotoxin limits, while national authorities establish regulations based on these guidelines [115].	Ensuring compliance with regulations requires robust enforcement mechanisms and regular updates to guidelines based on new scientific data. Regulation variation between countries can also complicate international trade and food safety efforts.
Food Safety Monitoring	Monitoring involves regularly testing food and feed samples to detect and quantify mycotoxin contamination [116].	Food safety authorities monitor mycotoxin levels using analytical techniques and surveillance programs targeting high-risk products and regions.	Effective monitoring requires access to reliable and sensitive analytical methods and resources for sample collection and testing. Ensuring consistent testing quality and managing large volumes of samples can be resource-intensive.
Enforcement and Compliance	Enforcement ensures compliance with mycotoxin regulations and prompts corrective actions for contamination [117].	Regulatory authorities inspect and enforce mycotoxin standards, with non-compliance leading to fines, recalls, or closures.	Effective enforcement requires coordination and adequate resources to ensure compliance among all stakeholders, especially in regions with limited infrastructure.

**Table 12:** Detection challenges.

	Issue	Challenges	Impacts
Sensitivity	Mycotoxins are often present at very low concentrations in food, making it challenging to detect them reliably. Analytical methods must be sensitive enough to identify trace amounts of mycotoxins to ensure accurate safety assessments [122].	Some mycotoxins have low natural abundance or are masked by matrix effects, which can interfere with detection. Analytical methods must be optimized to enhance sensitivity while minimizing false negatives.	Low sensitivity can result in underestimating contamination levels, potentially leading to unsafe food products reaching consumers.
Specificity	The specificity of detection methods is crucial to differentiate between mycotoxins and other compounds with similar chemical properties. Cross-reactivity with other substances can lead to false positives or inaccurate quantification [123].	Some methods, like immunoassays, may lack specificity and produce cross-reactivity with structurally similar compounds. Therefore, it is essential to ensure that methods can accurately target the specific mycotoxin of interest.	Lack of specificity can compromise the accuracy of results and lead to unnecessary regulatory actions or misinformed safety assessments.
Cost	Advanced analytical techniques, such as high-performance liquid chromatography–mass spectrometry (HPLC-MS), can be expensive due to equipment, reagents, and maintenance costs. This can limit their accessibility, especially in resource-limited settings [124].	High costs can restrict the frequency of testing and the number of samples analyzed, potentially leading to gaps in monitoring and an increased risk of undetected mycotoxin contamination.	Costs may limit the implementation of comprehensive testing programs, especially in developing regions with scarce resources.

**Table 13:** Control measure limitations.

Measures	Issue	Challenges	Impact
Pre-Harvest Control Measures	Pre-harvest measures, such as crop rotation and resistant varieties, are designed to reduce fungal contamination but may not always be practical or feasible [10].	Implementing crop rotation requires careful planning and may not be feasible for all crops. Resistant varieties may not always be available or fully protective.	Inadequate pre-harvest measures can lead to ongoing fungal contamination and mycotoxin production, especially in resource-limited regions.
Post-Harvest Control Measures	Post-harvest control measures, such as proper storage and processing techniques, are essential for managing mycotoxin contamination but may have limitations in effectiveness and feasibility [125].	Maintaining optimal storage conditions requires significant infrastructure, which may be lacking in some regions. Processing methods like heat treatments may not completely degrade all mycotoxins.	Inadequate post-harvest measures can lead to persistent mycotoxin contamination in stored food products, reducing the overall effectiveness of control strategies.
Chemical Treatments	Chemical treatments, such as the use of adsorbents or decontamination agents, can help reduce mycotoxin levels but are not consistently universally effective [126].	The effectiveness of chemical treatments can vary depending on the type of mycotoxin and the food matrix. Additionally, there may be concerns about potential residues or impacts on food quality and safety.	Variability in treatment effectiveness can limit the reliability of chemical methods in ensuring food safety and may require additional validation for different mycotoxins and food types.
Regulatory and Monitoring Approaches	Regulatory and monitoring systems are essential for ensuring compliance with mycotoxin limits but face challenges related to implementation and enforcement [127].	Regulatory guidelines vary between countries, causing inconsistencies in standards. Monitoring programs need substantial resources for sampling and testing, which may pose challenges in resource-limited settings.	Inconsistent regulations and inadequate monitoring can result in gaps in food safety oversight and an increased risk of mycotoxin contamination in the food supply.

**Table 14:** Research gaps.

Research Gaps	Need	Opportunity
Development of New Analytical Methods	While current analytical techniques such as HPLC, GC-MS, and ELISA are widely used, more advanced methods that offer improved sensitivity, specificity, and cost efficiency are needed.	Research into novel analytical techniques, such as portable sensors, lab-on-a-chip devices, or advanced mass spectrometry methods, could provide faster, more accurate, and cost-effective mycotoxin detection. Improved methods that can analyze multiple mycotoxins simultaneously or in real time would greatly enhance monitoring capabilities [126].
Innovative Control Strategies	Existing control strategies, including pre-harvest and post-harvest measures, are limited in effectiveness. Innovative approaches that can provide more reliable and scalable solutions are needed.	Research into biocontrol agents, such as beneficial microorganisms that inhibit fungal growth, or the development of novel chemical treatments that are both effective and safe, could offer new solutions for mycotoxin management. Additionally, integrating intelligent agriculture technologies, such as precision farming and remote sensing, may provide real-time data to optimize control measures [128].
Understanding Mycotoxin Interactions and Synergistic Effects	Many studies focus on individual mycotoxins, but there is limited research on the interactions and synergistic effects of multiple mycotoxins present in food.	Investigating how different mycotoxins interact and their combined effects on health could improve risk assessments and lead to more comprehensive control strategies. This includes studying the potential for additive or synergistic effects on toxicity and health outcomes [129].
Impact of Climate Change	Climate change can influence fungal growth and mycotoxin production, but the specific impacts on mycotoxin contamination and food safety are not fully understood.	Research into how changing climate conditions affect fungal populations, mycotoxin production, and crop susceptibility can help develop adaptive strategies and predictive models to better manage risks in varying environmental conditions [130].

**Table 15:** Key emerging trends.

	Trend	Impact
Advancements in Analytical Technology	New technologies, such as portable and field-deployable sensors, are emerging for rapid on-site mycotoxin detection. These devices can provide real-time results and are becoming increasingly affordable [131].	Portable sensors and devices can facilitate more frequent and widespread monitoring of mycotoxin contamination, particularly in developing regions or during critical stages of the food supply chain. These advancements improve the ability to detect contamination early and take appropriate actions.
Integration of Machine Learning and Artificial Intelligence	Machine learning (ML) and artificial intelligence (AI) enhance mycotoxin analysis by predicting contamination risks and optimizing control measures [132].	AI and ML algorithms can enhance predictive models for mycotoxin contamination, improve risk assessments, and optimize agricultural practices and monitoring systems. This integration can lead to more effective and targeted interventions.
Biocontrol and Natural Remedies	Research on biocontrol agents, like beneficial microbes and plant extracts, is increasing for environmentally friendly fungal inhibition and mycotoxin degradation [133].	The development and application of biocontrol agents offer a sustainable approach to managing mycotoxins. These natural remedies can reduce reliance on chemical treatments and contribute to more eco-friendly agricultural practices.
Enhanced Food Safety Regulations	Regulatory bodies continually update and refine guidelines and standards for mycotoxin levels in food and feed based on new research and emerging risks [134].	Enhanced regulations and standards can improve food safety and consumer protection. Continuous updates to regulatory frameworks ensure that they reflect the latest scientific knowledge and address emerging threats.
Global Collaboration and Data Sharing	Increased global collaboration and data sharing among researchers, regulatory bodies, and industry stakeholders are becoming more prevalent [135].	Collaborative efforts and shared data can improve the understanding of mycotoxin risks, enhance monitoring and control strategies, and promote the development of global best practices for mycotoxin management.

## Data Availability

No new data were created or analyzed in this study.
